# A Comprehensive Review of Viral Characteristics, Transmission, Pathophysiology, Immune Response, and Management of SARS-CoV-2 and COVID-19 as a Basis for Controlling the Pandemic

**DOI:** 10.3389/fimmu.2021.631139

**Published:** 2021-02-26

**Authors:** Chris R. Triggle, Devendra Bansal, Hong Ding, Md Mazharul Islam, Elmoubashar Abu Baker Abd Farag, Hamad Abdel Hadi, Ali A. Sultan

**Affiliations:** ^1^Department of Pharmacology, Weill Cornell Medicine-Qatar, Education City, Qatar Foundation, Doha, Qatar; ^2^Department of Health Protection & Communicable Diseases Control, Ministry of Public Health, Doha, Qatar; ^3^Department of Animal Resources, Ministry of Municipality and Environment, Doha, Qatar; ^4^Communicable Diseases Centre, Hamad Medical Corporations, Doha, Qatar; ^5^Department of Microbiology and Immunology, Weill Cornell Medicine, Cornell University, Doha, Qatar

**Keywords:** SARS-CoV-2, COVID-19, immune response, pathophysiology, drugs, COVID vaccines, transmission

## Abstract

COVID-19 emerged from China in December 2019 and during 2020 spread to every continent including Antarctica. The coronavirus, SARS-CoV-2, has been identified as the causative pathogen, and its spread has stretched the capacities of healthcare systems and negatively affected the global economy. This review provides an update on the virus, including the genome, the risks associated with the emergence of variants, mode of transmission, immune response, COVID-19 in children and the elderly, and advances made to contain, prevent and manage the disease. Although our knowledge of the mechanics of virus transmission and the immune response has been substantially demystified, concerns over reinfection, susceptibility of the elderly and whether asymptomatic children promote transmission remain unanswered. There are also uncertainties about the pathophysiology of COVID-19 and why there are variations in clinical presentations and why some patients suffer from long lasting symptoms—“*the long haulers*.” To date, there are no significantly effective curative drugs for COVID-19, especially after failure of hydroxychloroquine trials to produce positive results. The RNA polymerase inhibitor, remdesivir, facilitates recovery of severely infected cases but, unlike the anti-inflammatory drug, dexamethasone, does not reduce mortality. However, vaccine development witnessed substantial progress with several being approved in countries around the globe.

## Introduction

The highly infectious coronavirus disease 2019, COVID-19, is caused by the RNA virus, severe acute respiratory syndrome coronavirus 2 (SARS-CoV-2), which was first identified in Wuhan, China in December 2019 and declared a pandemic by the World Health Organization (WHO) in March 2020. By the end of 2020 COVID-19 had reached all continents including Antarctica sparing just a few Pacific Island nations, and daily increases can be monitored via accessing the Johns Hopkins Coronavirus Resource site (1). Consequently, the impact of the pandemic on healthcare is unprecedented stretching healthcare systems across the globe, as well as endangering healthcare professionals as one in seven has been infected (2). The swift identification and sequencing of the SARS-CoV-2 genome initiated a global program to develop vaccines as well as to investigate potential therapeutic agents. In this review we update our current knowledge of the genome structure of SARS-CoV-2 including mutational changes and implications on infectivity, transmission, pathophysiology, and host immune responses. We will discuss the impact of COVID-19 on the pediatric and elderly populations, assess the evidence for the effectiveness of therapeutics options, progress in the development of vaccines, and current regional public health measures to control the pandemic.

## Origin and Transmission of SARS-CoV-2

### Origin of SARS-CoV-2

**Figure 1:**
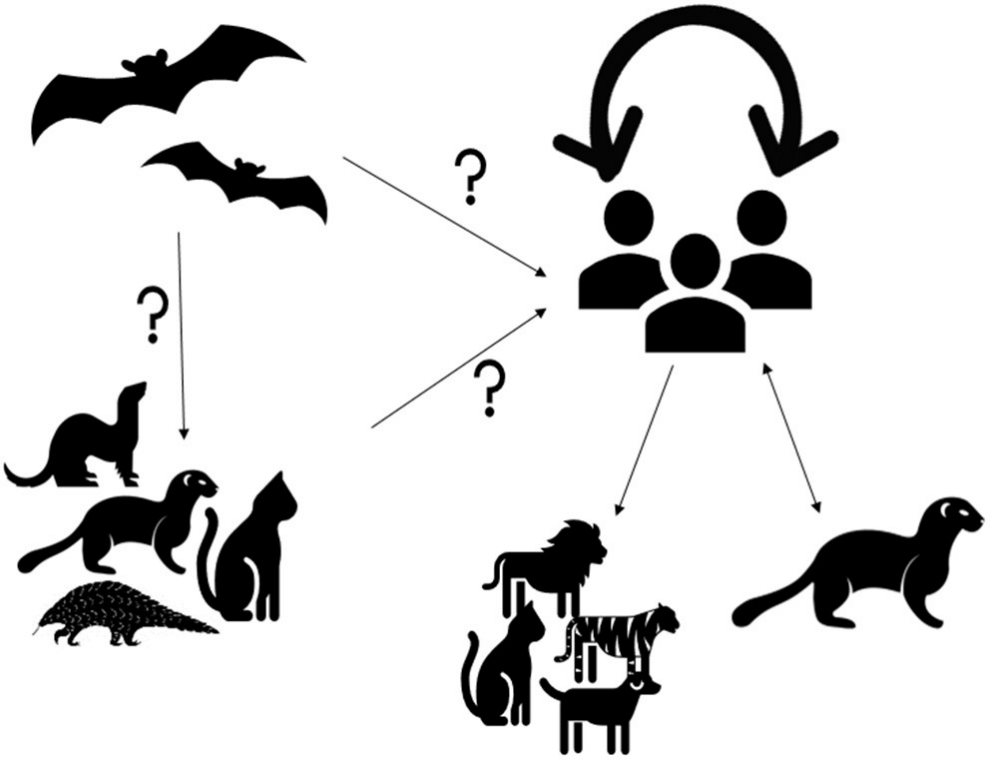
Origin and dynamics of SARS-CoV-2. SARS-CoV-2 is suspected to originate from bats (8, 9). It is unclear if the virus is transmitted to humans directly from bats or via intermediate hosts such as cats, pangolins, ferrets, minks or other animals. Infection with SARS-CoV-2 in cats, dogs, tigers, and lions were suggested to originate from human carriers (10). SARS-CoV-2 transmission from humans to minks and from minks to humans was detected in the Netherlands and Denmark (11, 12). However, human to human transmission remains the main route of transmission during the current pandemic (13).

Human epidemiological data links early spillover of SARS-CoV-2 to the Wuhan Seafood Market in China, where various livestock and species of wildlife and their products were sold (3). Most of the positive samples came from the Western part of the Huanan Seafood Market where animal facilities were located (4). In the absence of clear epidemiological evidence it was predicted that either the virus was introduced into the human population from an animal source from within the Huanan Seafood Market or alternatively an infected human could have introduced the virus into the market (5). Early reports were highly suggestive that SARS-CoV-2 jumped from bats via animal reservoir transmission, similar to that for SARS-CoV and MERS-CoV through civet cats and dromedary camels (4, 6–8). Based on the molecular similarities between SARS-CoV and the SARS-CoV-2, it can be assumed that the transmission route of the virus to humans involves an intermediate unidentified domestic, domesticated, or wild animal host (Figure 1). Coronaviruses are species-adapted where over time mutation of the viral genome can be adapted to new hosts, making transmission from one species to another rare. Nevertheless, some of the coronavirus species have shown a broad host range, such as SARS-CoV, MERS-CoV, and Bov-CoV (14). Although, coronavirus mutation rates are not as frequent compared to other viral families, RNA viruses are generally more susceptible to mutation compared to DNA viruses (15). Subsequently, with a significant mutation of the SARS-CoV-2 virus it could have been transmitted to humans through contact, handling or consumption of infected animals.

Genetic sequence data reveals that SARS-CoV-2 is a close relative of other species of coronaviruses found to be circulating in Rhinolophus bat (Horseshoe Bat) populations (9, 16). A 99% sequence similarity was detected between coronaviruses from Malayan pangolin and SARS-CoV-2 (4, 17). It has also been suggested that SARS-CoV-2 may have been transmitted to humans from snakes (4). When Egyptian fruit bats were infected with SARS-CoV-2 in a laboratory settings, these animals did not show signs of disease, but were able to transmit the virus to others (18). Experimental infection studies have also shown that ferrets and domestic cats were susceptible to infection by SARS-CoV-2 and could efficiently transmit the virus to uninfected animals (19–22). Other studies have shown that Golden Syrian hamsters, as well as cynomolgus and rhesus macaques, could be infected by SARS-CoV-2 and may show clinical signs (23, 24). Similarly, the Erasmus University Rotterdam research group found that rabbits are potentially susceptible (25). Dogs were also shown to be susceptible to the infection but appear to be less affected than ferrets or cats (19–21). Furthermore, previous natural infections with SARS-CoV-2 were detected in cats, dogs, lions, minks, and tigers (26–32). In addition, a new emerging strain of SARS-CoV-2 has been identified in minks, which were exposed to infected humans (33). The discovery was made in Denmark where the variant was observed in 12 human cases and which has since been labeled as “cluster 5,” with its genetic makeup yet to be fully understood (11). This information elucidates the notion that minks could serve as a reservoir for SARS-CoV-2 and pose a risk for a spill-over to human populations as reported in The Netherlands (34). It is not uncommon for human to animal transmission to occur which can lead to genetic mutations of the virus. As a consequence, the WHO has encouraged all countries to strengthen their biosafety and biosecurity measures to reduce the zoonotic risks of events in relation to SARS-CoV-2, including infection prevention and control measures of farms and animal industry (33, 35). In January 2021, the WHO tasked an international scientific committee of experts in field of epidemiology and infectious diseases to investigate the origin of the COVID-19 pandemic in China using a scientific and transparent approach not only for identifying the origin of the current pandemic but for the future of global health security to manage emerging infectious threats (35).

### SARS-CoV-2 Transmission

SARS-CoV-2 is a pneumotropic virus that spreads from person-to-person predominantly through respiratory secretions including droplets generated through coughing, sneezing or even via talking. Transmission via personal contact, contaminated surfaces or fomites also contributes, particularly in settings where non-pharmaceutical interventions (NPIs) such as hand hygiene, masks and appropriate social distancing are not consistently applied (36, 37). In indoor settings with inadequate ventilation SARS-CoV-2 remains highly infectious as aerosols potentially for hours and can travel for tens of meters before landing on surfaces, where the virus can survive for up to 6 days (38–40). Since the beginning of the pandemic various NPI mitigation measures have been implemented, albeit inconsistently, to hamper the spread of the disease, including contact tracing, avoidance of mass gathering and community lockdowns. The danger of airborne transmission calls for additional preventative measures such as social and occupational health measures to ensure that buildings are well-ventilated and not overcrowded. In early July 2020, 239 scientists from more than 30 countries submitted an open letter to the WHO urging the organization to highlight the potential high risk of an airborne spread (41).

## SARS-CoV-2 Genome Structure: Role in Infectivity and Virulence

SARS-CoV-2 is a single-stranded, positive-sense, non-segmented enveloped RNA virus of ~ 29.9 kB in size with a diameter of 50–200 nm (42). Structurally, it has a double-layered lipid envelope, including spike (S) glycoprotein, envelope protein, membrane glycoprotein, and nucleocapsid protein (43, 44). The viral spike glycoprotein has a receptor-binding domain (RBD) for the interaction with host cell receptors (45).The membrane glycoprotein is responsible for the assembly of viral particles (46). The envelope protein is reported to play a role in pathogenesis as it interacts with the tight junction related protein, named; Protein Associated with Caenorhabditis elegans Lin-7 protein 1 (PALS1) (47). The nucleocapsid protein, which is a phosphoprotein, packs the viral genome into a ribonucleoprotein complex and plays a role in viral genome replication and the cell-signaling pathway (48).

**Figure 2:**
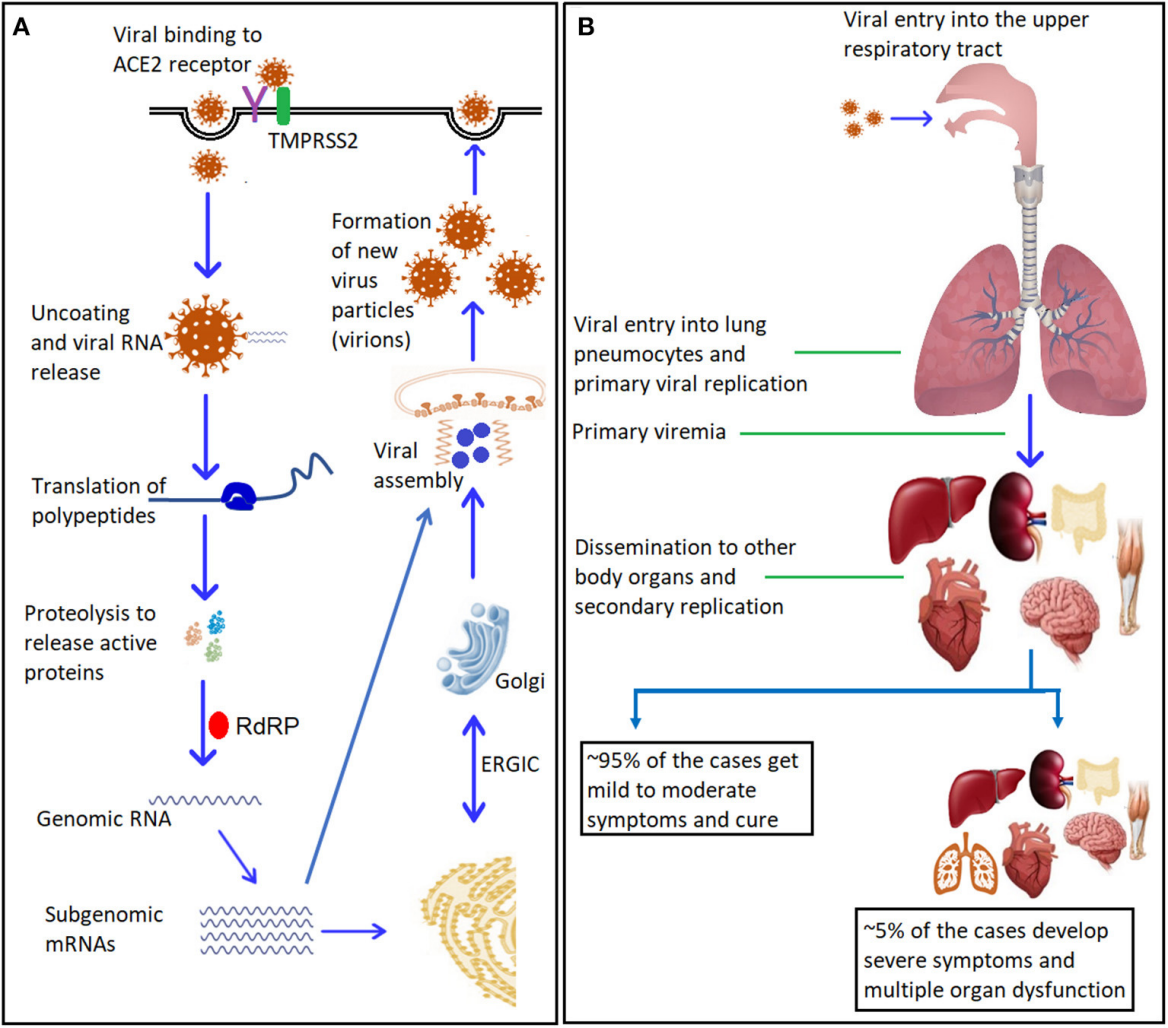
SARS-CoV-2: mechanism of entry, replication, and dissemination. **(A)** Entry and replication of SARS-CoV-2 inside the host cells. The virus spike glycoprotein binds to the cellular receptor ACE2. This binding induces conformational changes at the receptor binding domain (RBD) that expose co-receptors to bind with transmembrane protease serine-2 (TMPRSS2), with the help of cellular endosomes it facilitates viral internalization via endocytosis. Internalization results in uncoating of viral genomic RNA into the cytoplasm. Genomic RNA binds to the host ribosome, which leads to formation of polyproteins that contain transcription complex, including the viral RNA-dependent RNA polymerase (RdRP). The RdRP generates viral genomic RNA and several subgenomic mRNAs, which will be translated into relevant viral proteins. The translated proteins translocate into endoplasmic reticulum (ER) membranes and transit through the ER-to-Golgi intermediate compartment (ERGIC), where interaction with encapsidated, newly produced genomic RNA results in budding into the lumen of secretory vesicular compartments. The newly formed viral particles (virions) are subsequently released out of the cells via exocytosis. **(B)** Viremia and dissemination into body organs: Initial replication takes place in the upper respiratory tract, followed by the migration of the virus to the lungs. A primary viremia occurs after establishment of infection and replication in the lung pneumocytes. This viremia disseminates the virus throughout the body via blood stream where another cycle of viral replication takes places and ensuing viremia leads to further dissemination (for more details and references see section SARS-CoV-2 Genome Structure: Role in Infectivity and Virulence and Replication Cycle and Pathophysiology of the review). ACE2, Angiotensin-converting enzyme 2; RdRP, RNA-dependent RNA polymerase. ER, Endoplasmic reticulum; ERGIC, ER-Golgi Intermediate Compartment.

**Figure 3:**
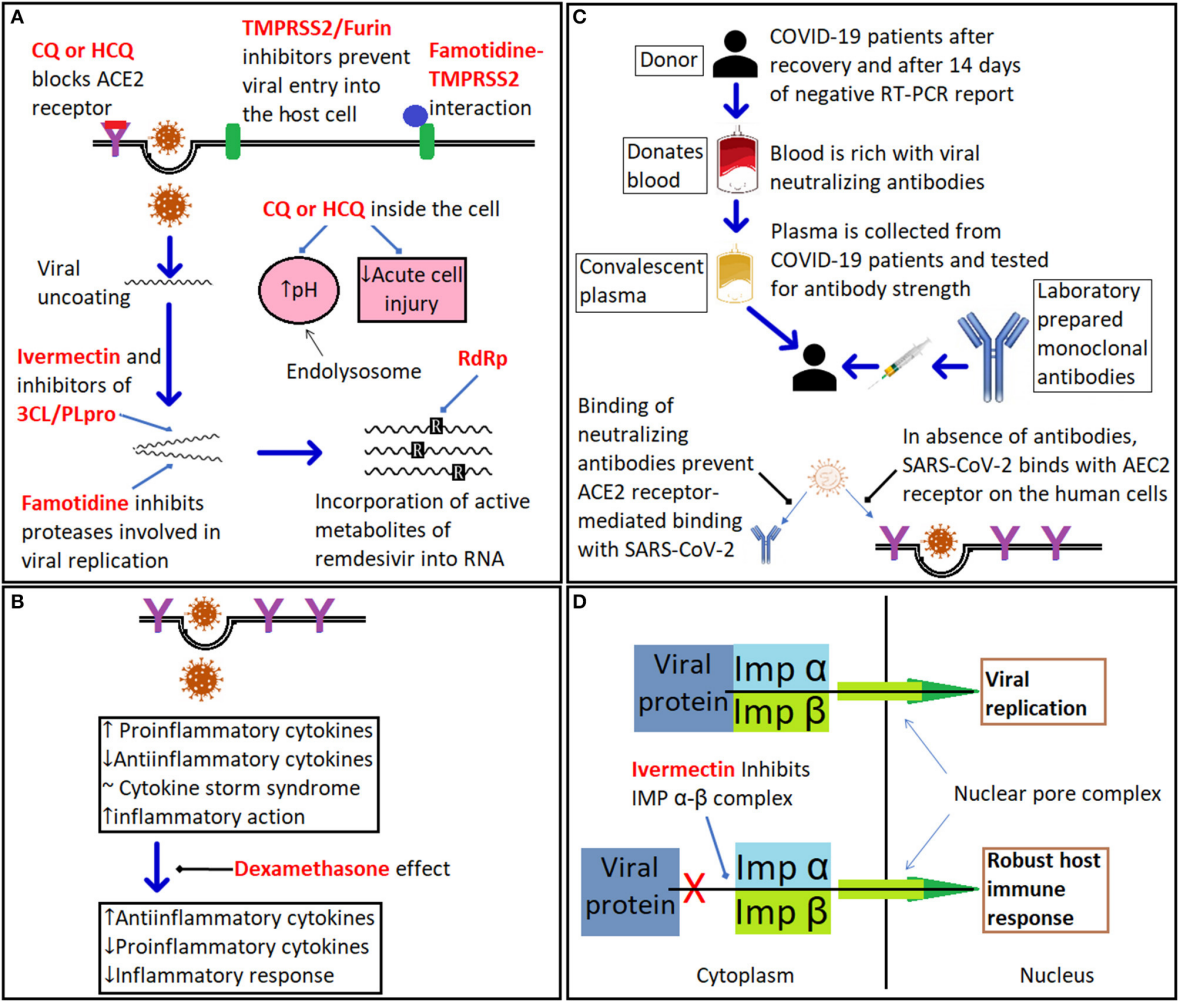
The mechanism of action of different therapeutics against COVID-19. **(A)** illustrates the mode of action of drugs targeting COVID-19 including chloroquine (CQ) and hydroxychloroquine (HCQ), which have multiple putative sites of action: (i). ACE2 receptor for SARS-CoV-2; (ii). increasing the pH of the endolysosome; and (iii). suppression of the immune response. Sites of action of TMPRSS2 inhibitors such as camostat, famotidine, and furin inhibitors are shown; famotidine is also a putative inhibitor of the 3CL/PLpro proteases; ivermectin is a putative TMPRSS2 inhibitor that also inhibits the importin (IMP) α-β complex and viral replication; while remdesivir inhibits viral RNA polymerase. **(B)** Dexamethasone suppresses expression of pro-inflammatory cytokines. **(C)** Summary of role of convalescent plasma and monoclonal antibody therapy. **(D)** Ivermectin inhibits the heterodimeric importin (IMP) α/β complex via binding directly to IMPα preventing nuclear import of key viral proteins (for more details and references, see section COVID-19 therapeutics).

The SARS-CoV-2 virus enters host cells via hijacking physiological ACE2 receptors where it uses the Spike (S) glycoprotein for their attachment (Figure 2) followed by internalization (49). The spike glycoprotein has two subunits, S1 and S2. The S1 subunit consists of the receptor-binding domain (RBD), which binds with the receptor-binding motif (RBM) of cell surface receptor, while the S2 subunit, mediates fusion with the host cell membrane (50, 51). The spike protein is cleaved by host proteases (located on S2 sub-unit) to make necessary conformational changes for membrane fusion (50, 52). The type II transmembrane serine protease (TMPRSS2) is the main host protease that mediates S protein activation and initial viral entry into primary target cells (53, 54). Camostat mesylate, an inhibitor of serine protease TMPRSS2, blocks the entry of coronaviruses into the cell indicating the its important role in priming the S glycoproteins thus initiating infection (54). Furin is another host protease, which plays an important role in cellular pathogenesis of COVID-19 (55) (Figure 3).

One of the most comprehensive approaches in understanding genetic determinants of viral virulence as well as identifying host specific immune adaptations, is the study of the evolution of the virus through whole genome sequencing (WGS). This advanced process resulted in rapid sequencing of thousands of SARS-CoV-2 genomic isolates from different parts of the world, following deciphering the foremost COVID-19 strain from China published in early January 2020 (56).

Mutational hot spots in the SARS-CoV-2 virus include non-structural proteins (NSPs): NSP2 and NSP3, S protein, and RNA-dependent RNA polymerase (RdRp) (57), and mutations in NSP2 and NSP3 proteins and S protein are key contributory factors in virulence and differentiation mechanisms of SARS-CoV-2 that affect how it spreads and evolves (58). Genomic analysis revealed that mutations in SARS-CoV-2 accumulate at a significantly slower rate when compared with other extensively studied RNA viruses such as influenza and HIV (59).

Structural sequencing analysis of SARS-CoV-1 spike glycoprotein and SARS-CoV-2 spike glycoprotein reveals 76% amino-acid sequences homology (55, 60). Moreover, both SARS-CoV-1 and SARS-CoV-2 shares eight conserved binding positions and six semi-conserved positions in their S protein domains (55, 61). Therefore, the binding efficiencies of the spike glycoproteins of both SARS-CoV-1 and SARS-CoV-2 are similar to each other, however, the conservation and semi-substitution in spike glycoprotein have somehow made the SARS-CoV-2 more adaptable to the ACE2 receptors, thereby enhancing transmission (55). Furthermore, using comparative genomic techniques, Gussow et al. identified important features that differentiate SARS-CoV-2 from SARS-CoV and MERS-CoV (62). These features include enhancement of the nuclear localization signals of the nucleocapsid proteins through which the spike glycoprotein inserts and appears to be associated with higher case fatalities as well as inter species transmission from animals to humans (63). Based on ongoing genome sequencing of SARS-CoV-2, multiple SARS-CoV-2 variants have been reported are circulating globally (64). Mutations of the spike proteins are of fundamental importance because of their role in host cell entry as well as being the main target for immune neutralizing antibodies and vaccine development. For instance, a change of an amino acid on spike protein from aspartate to glycine at position 614 (D614G) was first described in March 2020, and by June 2020 it had been reported worldwide. Although there was an initial concern that the D614G mutation confers enhanced transmission, this was subsequently refuted (59, 63). Several new variants emerged in the fall of 2020, most notably:

a) A new variant of SARS-CoV-2 (coined VOC 202012/01 or B.1.1.7) emerged in late summer to early Autumn 2020 in United Kingdom (65, 66). This variant has since been detected in numerous countries around the world (67, 68) and has 17 mutations, eight of which are in the spike protein (69, 70). Based on mathematical models, calculations by the COVID-19 Genomics UK consortium suggest that B.1.1.7 variant might be up to 70% more transmissible than the original virus (67, 71),
b) A non-related variant of SARS-CoV-2 (coined N501Y.V2 or B.1.351) emerged in South Africa independent from that in the UK variant, which bears mutational similarities to the latter (66). Cases attributed to this variant have been detected outside of South Africa (66). The N501Y.V2 variant seemed to improve the virus's binding abilities, which could lead to enhanced transmissibility (72),
c) A third new distinct variant of SARS-CoV-2 emerged in Nigeria, which did not share any of the unique mutations of the aforementioned variants (66, 67, 73).

Similar to other RNA viruses, SARS-CoV-2 is evolving and mutating all the time, thus such variants are completely expected, although coronaviruses in general tend to mutate more slowly. From recent reports current evidence suggests that the newly detected SARS-CoV-2 variant in the UK offers evolutional advantages in term of transmission rather than virulence (68, 74). While awaiting detailed confirmation, a pre-print article highlighted that an engineered SARS-CoV-2 with the N501Y mutation, one of many mutations present in the new emerging viral variants B.1.1.7 and 501.V2, can be neutralized by the sera of COVID-19 vaccine recipients (75).

As a consequence, the future course of the COVID-19 pandemic will be determined by the virological diversity, epidemiological spread as well as the clinical characteristics of these variants. Whether such variants confer biological advantages over the host will determine infectivity and virulence and ultimately the efficacy of vaccines as well as targeted therapies.

## Replication Cycle and Pathophysiology

The pathophysiological sequalae of infection with SARS-CoV-2 is illustrated in Figures 2A,B. The primary route of entry for SARS-CoV-2 is through the upper respiratory tract or facial mucosal surfaces. Viral particles gain access into cells by binding to ACE2 receptors followed by receptor-mediated endocytosis, similar to mechanisms used by the closely related virus, SARS-CoV (76). This entry mechanism has been confirmed experimentally when anti-ACE2 monoclonal antibodies were deployed into cell cultures blocking viral entry (77). Uncoating of viral genetic material and protein allows for the release of the RNA and translational proteins including RNA dependent polymerase followed by viral transcription and assembly and subsequent viral shedding to complete replication cycle (78).

In humans, the primary physiological function of ACE2 is to convert the peptides angiotensin I and II into angiotensin 1–9 and angiotensin 1–7, which provide protective cardiovascular functions through mechanisms that include vasodilatation and control of endothelial permeability (79). Infection with SARS-CoV-2 results in a reduction in the levels of ACE2 and disruption in the Renin Angiotensin Aldosterone System (RAAS), which amplifies signaling through the angiotensin II pathway, resulting in potentially severe inflammatory and circulatory dysfunction (79). In addition to its expression in respiratory cells including upper airways, ACE2 receptors have also been isolated from the endothelium, gastrointestinal and renal tissues hence explaining the multisystem presentations (80). The comparatively high prevalence of COVID-19 in individuals with hypertension or diabetes raised the concern of the role of the ACE2 receptors in this vulnerable population particularly given that a significant proportion of them might be concomitantly treated with ACE inhibitors (ACEIs) or angiotensin II receptor blockers (ARBs). There was initial concern that the concomitant treatment of COVID-19 patients with ACEIs or ARBs would enhance ACE2 expression and hence susceptibility to infection and pathophysiological sequelae, but fortunately, this has not been supported by clinical evidence (28). Arguably, because of the beneficial effects of ACE2 mediated pathways in offsetting the pathophysiological effects of RAAS pathway activation and thereby providing cardiovascular and renal protection ARBs and ACEIs may prove beneficial in patients with COVID-19 (81). To address these questions, data from clinical trials to determine whether ACEIs, or ARBs, are beneficial in COVID-19 in both inpatient and outpatient settings (identifiers: NCT04311177, and NCT04312009, respectively) are expected during 2021.

Although the major clinical manifestations of COVID-19 were immediately identified as primarily a respiratory disease with multisystem involvement and spectra of symptoms, the distinct underlying mechanisms that lead extrapulmonary presentations of COVID-19 have not been fully established (82). A systematic review of 18 studies with an estimated cumulative 15,000 COVID-19 cases revealed that the prevalence was highest amongst patients with hypertension (~23%), diabetes (~11.5%), cardiovascular disease (CVD) (~10%) as well as chronic pulmonary and kidney diseases (83). Furthermore, a higher level of expression of ACE2 in adipose tissue together with a hyper inflammatory state prevalent in obese individuals could increase the risk of a cytokine storm and end organ damage (84).

While exposed patients can be asymptomatic or develop mild to moderate symptoms, around 20% suffer from severe disease that requires oxygen supplementation and, sometimes, critical care with ventilator support (85). There have been reports of long-term respiratory sequelae such as impairment of lung functions due to fibrosis, long-term neurological dysfunctions including variable loss of taste and olfactory senses, neuropathies, as well as memory dysfunction (82). From the histopathological examination of autopsy cases, destruction of alveolar structures denotes direct viral cytopathic effects (86). During the pandemic, it became apparent that arterial and venous thromboembolism are characteristic features of the disease indicating that endothelial inflammation is an integral severity contributor to the progression of COVID-19 leading to a state of coagulopathy and thrombotic or hemorrhagic tendencies manifested as pulmonary embolisms, cardiovascular or CNS strokes particularly in patients with severe disease (87–90). In support of these observations, occlusive and non-occlusive acute coronary syndromes resulting in myocardial infarction have been observed in patients with minimal cardiac risk factors including considerably younger patients (79). These observations led to the importance of assessing the role of anticoagulation and antiplatelet therapy in COVID-19 where guidelines continue to evolve (91–94). Current guidelines do not support the routine prophylactic use of anti-coagulant/anti-platelet therapy in non-hospitalized patients unless clinical indications dictate otherwise. Furthermore, it has been recommended by different COVID-19 management guidelines that hospitalized non-pregnant adults with COVID-19 should receive prophylactic doses of anticoagulants such as low molecular weight heparin. What is the optimal anticoagulation approach in critically ill patients that require intensive support is unclear as three trials studying whether therapeutic doses reduce progression toward organ support have been halted since intervention therapy was observed to be ineffective for organ protection: (1) The Randomized, Embedded, Multi-factorial Adaptive Platform Trial for Community-Acquired Pneumonia (REMAP-CAP); (2) Therapeutic Anticoagulation; Accelerating COVID-19 Therapeutic Interventions and Vaccines-4 (ACTIV-4); Antithrombotic Inpatient; (3) Antithrombotic Therapy to Ameliorate Complications of COVID-19 (ATTACC) (95).

One of the most intriguing phenomena of the clinical manifestations of the pandemic disease is the selective development of the immune mediated COVID-19 cytokine storm (CCS). The clinical condition develops toward the end of the first week following infection, probably the outcome of both specific pathogen and host related factors. Due to mechanisms, yet to be fully understood, a subgroup of patients instead of suppressing viral replications toward viral clearance and recovery, mounts an overwhelming catastrophic hyper-immune pro-inflammatory response characterized by the massive production of cytokines such as Tumor Necrosis Factor alpha (TNF-a), interleukins particularly IL1, 6, and 8. This hyper-immune pro-inflammatory response produces to ominous multisystem manifestations including prominent vascular permeability causing secondary cardiovascular instabilities, a hypercoagulable state leading to serious multitudes of venous and arterial thromboembolism, tissue inflammation and destruction culminating into ominous acute respiratory distress syndrome (ARDS) and frequently lethal consequences (96). The phenomena have been recognized early during the pandemic which instigated suppression of the harmful effects of the immune responses through various suppressive mechanism including steroids, anticoagulation, anti TNF treatment as well as interleukins monoclonal antibodies inhibitors including the IL-1 inhibitor, anakinra, and the IL-6 inhibitor, tocilizumab Eventually steroids proved to be beneficial as documented in the landmark RECOVERY trial which has been reciprocated (97). Despite conflicting efficacy results for tocilizumab from cohort and randomized clinical trials, evidence of significant benefits have been contradicted by data from larger randomized studies including met analysis suggesting that additional studies are required (98–100).

## Host Response to Covid-19 Infection

Understanding the host immune response to SARS-CoV-2 infection is critical to understanding the pathogenesis of COVID-19, clinical manifestations, passive or active mediated responses such as monoclonal antibodies and convalescent plasma therapies, as well as vaccine development against the disease. The host immune response to COVID-19 determines susceptibility to the progression of infection as well as being a major determinant of recovery orchestrated by coordinated B and T cell responses (101). Furthermore, data concerning the durability of immunity to SARS-CoV-2 infection can assist in the continued development of successful vaccines and therapeutics for control of COVID-19.

Our current understanding of SARS-CoV-2 immunity is based mainly on previous experiences with other corona viruses including SARS-CoV and MERS-Co V viral infections, supplemented with studies of patients who were infected and recovered from SARS-CoV-2 infection (102, 103). Data on the biological (e.g., age, sex, and body mass index) and genetic factors responsible for disease severity to SARS-CoV-2 are just emerging. For example, neutralizing antibodies, and responses from CD4^+^ and CD8^+^ T cells, might be associated with COVID-19 severity, with age being a risk factor (104). So far, current data indicate that innate immune and adaptive immune responses, including antibody production and T-cells, are involved in response to infection by SARS-CoV-2 and the pathogenesis of COVID-19.

### Innate Immune Response

Based on previous knowledge of the innate immune response to other coronaviruses the conserved mechanisms of innate immune signaling can be seen in patients infected with SARS-CoV-2 (105–107). Being an RNA virus, the SARS-CoV-2 innate immune response is triggered by pattern recognition receptors (PRRs) such as Toll-Like Receptors (TLRs) or Retinoic acid-inducible gene I (RIG-I)-like from the components receptors (RLRs) that enable to detect the virus (108). This elicits a downstream signaling cascade, which leads to secretion of cytokines such as type I/III interferons (IFNs), tumor necrosis factor alpha (TNF-a), interleukin-1 (IL-1), and IL-6 among other cytokines. These cytokines will induce antiviral activity in the host cells, which subsequently induces adaptive immune responses. Additionally, if released early as well as being properly localized, IFN-I can effectively control SARS-CoV infection (109). Data from *in vitro* studies suggest that SARS-CoV-2 is susceptible to IFN-I/III pretreatment (110–113). Other molecules of the innate immune system such as lymphocyte antigen six complex loci E (LY6E) and the IFN-induced transmembrane family (IFITM) proteins can inhibit SARS-CoV-2 (114–116).

However, this innate immune response is reported to be impaired during SARS-CoV and MERS-CoV infection by their non-structural proteins, which affects the overall cytokine production (117, 118). For example, SARS-CoV-1 inhibits IFN-I both *in vitro* and *in vivo* (119–122). Studies have shown that SARS-CoV-2 lack strong type I/III IFN signatures, and patients with severe COVID-19 displayed impaired IFN-I signatures as compared to mild and moderate cases (110, 123).

### Humoral Immunity

From previous experience of emerging infectious diseases, neutralizing antibodies have been identified as being pivotal immune components against viral infections. The major role of neutralizing antibodies is antigen binding and interaction with cells bearing Fc γ-receptors to modulate subsequent targeted immune responses.

Similar to SARS-CoV infection (124), humoral immune response to SARS-CoV-2 infection is mediated by antibodies that are directed to viral surface glycoproteins, mainly the Spike (S) glycoprotein and the nucleocapsid protein (125, 126). These antibodies are detectable approximately 6 days after RT-PCR confirmation of infection, and those directed against spike receptor-binding domain (RBD) demonstrate neutralizing ability and thus, viral clearance and prevention of infection (127). Following acute SARS-COV-2 infection, the time lag for the appearance of early IgM and late IgG antibodies ranges between 6 and 28 days being longer in milder cases (101, 128, 129). IgG, IgM, and IgA antibodies responses to the SARS-CoV-2 cysteine-like protease have also been reported in patients with COVID-19, and these responses correlate with antibody titers to the nucleocapsid protein (130). It is likely that viral clearance and recovery require coordinated B and T cell function but what is not clear is if it provides long-lasting immunity and protection from reinfection, which is crucial when considering the variable immune responses, and the prospects of protective vaccination (101). In a cohort study of COVID-19, detectable antibodies were observed in <40% patients within 1 week and increased within 2 weeks to 100, 94.3, and 79.8% for both or either, or IgM or IgG, respectively (63, 129). A longitudinal study looking at the kinetics of antibodies directed against the spike protein in patients with COVID-19 found that IgA antibodies were produced in the first week of the infection and peaks after 20–22 days, whereas IgM antibodies titers reached a peak level after 10–12 days and subsequently started to disappear 18 days after the onset of symptoms (131). Another study assessing the IgG responses to spike glycoprotein in COVID-19 patients found that IgG titers increased in the first 3 weeks after the onset of symptoms and started to decrease by 8 weeks (132).

Furthermore, data indicating that there is a rapid decrease, with 2–4 months, in the IgG antibodies responses directed against the viral RBD and nucleocapsid proteins in patients with mild COVID-19, suggesting that humoral immunity against SARS-CoV2 infection might not be long-lasting in patients with mild disease (133, 134).

On the other hand, a prospective study of patients with severe COVID-19 disease, a prospective study found significantly higher IgM and IgG antibodies titers in patients with severe disease in comparison to those without (135). Two of the major concerns regarding the current pandemic are: a) the possibility of reinfection and b) the longevity of protective vaccinations. In the case of infection from SARS-CoV-1 virus, IgG antibodies were maintained for 2 and 3 years in 90 and 50% of the patients, respectively (101, 102). Similarly, in seasonal coronavirus 229E infections, nasal IgA were detected 1-year post-exposure, despite undetectable serum IgA antibodies (101, 136). Current evidence suggests that, IgM and IgG antibody levels against SARS-CoV-2, may be maintained for 7 weeks (137) with at least 80% reported in cases following 49 days post infection (63, 137). Of importance is a report that anti-spike or anti-nucleocapsid antibodies in response to Covid-19, last at least 6 months, and was associated with a reduced risk of SARS-CoV-2 reinfection in the ensuing 6 months (138). Additional longitudinal and large cohorts' studies of patients with COVID-19 are vital to understand mounted and fluctuations in immune dynamics over time as well as the duration of protection by functional neutralizing antibodies and potential for re-infection (63, 101, 137, 139).

Interestingly, host immune response provides a potential therapeutic option through harvesting recovered patients' convalescent plasma (CP). Despite encouraging anecdotal data from cohort studies of the beneficial role of CP in the management of COVID-19, this therapeutic approach is facing challenges including the availability of suitable donors to match the rising number of patients, inefficiency based on cost-effectiveness as well as concerns of whether there is a lasting protective effect (140). As such, the current paucity of supportive evidence for CP make it less widely utilized (141). Distinctively, there may be differences amongst patients' subgroups and at what stage initiation of therapy is most beneficial as reflected in the positive data with CP from an RCT of 160 elderly patients with early symptomatic disease, suggesting that the benefits of CP in term of reducing disease progression are linked to waning of the immune response with age (142).

### Cell Mediated Immunity

Recognition of SARS-CoV-2 antigens by pre-existing and cross-reactive T cells generated during previous infection with human coronaviruses might contribute to the frequent presence of T cells reactive to SARS-CoV-2 in patients with COVID-19 (143). A strong T cell immune response has been demonstrated in convalescent people with asymptomatic or mild COVID-19 (144).

A fundamental role of T-cell mediated immunity was shown through a response against SARS-Cov-2 demonstrated by CD4+ T helper cells interacting with CD8+ T cells, along with natural killer cells, to drive the cytotoxic response to kill the infected cells (101, 145). Grifoni et al. (101) used HLA prediction algorithms and peptide mega-pools to identify SARS-CoV-2-specific T cells in 10 patients with COVID-19 and 11 healthy, unexposed control participants. Virus-specific CD4^+^ T-cell responses were detected in seven (70%) patients with COVID-19 and virus-specific CD8^+^ T-cell responses were detected in all 10 patients with COVID-19, further indicating that most individuals can develop T-cell responses to SARS-CoV-2. The CD4+ T-cell response predominantly consisted of T-helper-1 (Th1) cells, characterized by high concentrations of IFN-γ secretion directed against both structural (spike, nucleocapsid and membrane) proteins and non-structural proteins (146, 147). CD8^+^ T-cell responses specific to SARS-CoV-2 produced IFN-γ and tumor necrosis factor (TNF)α, also reflective of Th1 response.

During the COVID-19 pandemic, one of the early features is the noticeable overall reduction in total lymphocyte count, particularly CD4+ and CD8+ T cells, which correlates with disease severity as well as the development of critical complications (101, 148). Similarly, the role of type I interferons (type 1 INFs) in COVID-19 has been highlighted when observations showed that 10% of patients with life-threating pneumonia have neutralizing autoantibodies against type I IFNs suggesting a potential therapeutic role for IFNs (149). Furthermore, studies have shown that poor outcomes can be predicted in cases where there is a decrease in levels of CD8+ T cells and B cells or an increase in CD4+/CD8+ ratio (101, 145). It has been reported that the increase in T-cell responses in SARS-Co-V-2 infections is correlated to the severity of the disease (101, 150).

Both CD4^+^ T-cell and CD8^+^ T-cell responses occur in most patients infected by SARS-CoV-2 within the first 2 weeks after the onset of symptoms and produce mainly Th1 cells. The frequency of CD4 +T cells targeted to the spike protein correlates with neutralizing antibody titers (101), which may suggest that the T-cell response might be different among COVID-19 patients with different disease severity (144, 151).

Overall, several studies of COVID-19 patients showed that both humoral and cellular immunity are involved in the pathogenesis of COVID-19. Better understanding of the host immune responses and the pathogen immune evasion strategies will help in the development of effective vaccines against COVID-19 and hopefully a better future.

## COVID-19 in Children

Currently, the burden of COVID-19 in children has not been determined accurately because of the high prevalence of asymptomatic cases, and the lack of consensus on case definitions for screening, testing and disease severity in children (152). Additionally, febrile and respiratory illnesses are very common during early childhood, and, as a consequence, the diagnosis of COVID-19 may be easily overlooked (153).

The majority of published studies have concluded that children represent 1–2% of diagnosed COVID-19 infections (154). The median age of the diagnosed children ranges from 3.3 to 11 years in different reports (154, 155). Typically following exposure to infected family members, children are predominantly asymptomatic or experience milder symptoms compared to adults (156–158). This low prevalence can be attributed to scarcity of clinical symptoms, higher testing thresholds, or lower reporting of cases (152).

In comparison to infected adults and older children, younger children with mild/moderate COVID-19 are likely to have higher nasal viral loads, giving way for a silent spread of the virus especially with sequential opening of schools, ultimately a potential indicator for second and third waves (159). In contrast, some studies negated the role of children in viral transmission to adults in a sharp contrast to the axiomatic concept of transmissions of other related respiratory viral infections (33, 157). The low spread of infections amongst children might be explained by the precautions taken by public health authorities such as closure of schools, and public facilities as well as the lockdown of entire communities. Since these measures are being gradually lifted, an increase in the number of infections might be expected, with a report from the United States revealing a 90% increase in COVID-19 infections among children (160).

A large-scale study of over 2,000 pediatric patients in China, revealed that 94.1% of children with confirmed/suspected COVID-19 were asymptomatic or showed mild to moderate symptoms, 4.4% of these were completely asymptomatic while approximately 6% became severely or critically ill (161). This may explain the observed low mortality of <1% of all deaths (160). Nevertheless, it remains enigmatic if an immature immune response plays a protective role against the development of severe clinical manifestations in children (71, 162).

Regarding the clinical presentation of COVID-19 in children, respiratory symptoms are common, followed by fever and gastrointestinal symptoms (156, 157, 163) while the rate of children with critical illness ranges from 0.4 to 9% of confirmed cases, probably reflecting population bias since some reports include mainly patients diagnosed at hospitals (156, 157, 163). In various reports, almost 50% of the children admitted to the ICU had an underlying medical condition (156, 157, 164).

A specific matter of concern is the comparatively high incidence of multisystem inflammatory syndrome (MIS) reported in children diagnosed with COVID-19 (165–168). MIS manifests with fever, elevated inflammatory markers and organ dysfunction not attributed to another infectious cause (168). The most common presentations of MIS in children are fever, headache, along with gastrointestinal, cardiovascular, and respiratory symptoms mucocutaneous involvement (skin rashes and non-purulent conjunctivitis) as well as limb edema (168–170). Raised inflammatory markers such as neutrophilia, elevated C-reactive protein and elevated ferritin are frequent, and, in addition, thrombocytopenia, lymphopenia, elevated troponin and elevated D-dimer and fibrinogen have been observed in children with MIS (171). MIS can take a severe course requiring ICU admission and mechanical ventilation (168–173). A large cohort study reported that 2% of MIS patients died and underlying medical conditions were reported in 50% of children who died with MIS (169, 170).

MIS in children might be a late complication of COVID-19 because the median interval from the onset of COVID-19 symptoms to the onset of MIS is 25 days, and the higher rate of positive serologic test compared to nasopharyngeal RT-PCR (167, 174). Several pathophysiological mechanisms have been suggested as underlying causes of MIS in children. Such mechanisms include formation of immune complexes, viral mimicry, and host immune cell activation due to viral superantigen sequences (175) Treatment in children infected with SARS-CoV-2 consists mainly of supportive care, including oxygen and advanced respiratory support, hydration, and antipyretics (176, 177).

In addition to supportive care, children diagnosed with MIS were treated with intravenous immune globulin, glucocorticoids, IL-1 and IL-6 receptor antagonists as well as TNF-α antagonists (173).

## COVID-19 in Elderly Population

Elderly individuals infected with SARS-CoV-2 virus are at greater risk of developing severe infection and complications, leading to increased morbidity and mortality rates (37, 88, 178). A large scale study involving 72,314 patients from China reported that case fatality was 8.0% in patients aged 70–79 years and 14.8% in patients' aged ≥80 years (179). Another study from Europe reported 48% mortality in patients' aged ≥85 years (180). A demographic study measuring the contribution of age distribution of COVID-19 cases to the case fatality rates (CFRs) across nine countries, found that after age standardization, the highest CFRs were observed in Italy, Spain and the Netherlands, while the lowest CFR occurred in Switzerland, France, the USA and Germany (181). Sudharsanan et al., suggested that several factors such as the health of the population, the time of detection of COVID-19, the quality of care provided to patients and the state of preparedness within healthcare systems, could explain the differences in the CFRs across these countries (181).

Elderly patients usually have chronic medical illnesses and are likely to have severe or critically severe comorbidities. They might also show atypical symptoms without fever or cough and multiple organs dysfunction. Furthermore, most elderly patients at the emergency department present with atypical presenting illnesses including falls and may lack specific COVID-19 underlying symptoms (182, 183).

Several other studies have also suggested that elderly patients may not exhibit typical symptoms, such as fever or cough, when compared to young patients infected with SARS-CoV-2 (184). Fever in the elderly patients might be masked and COVID-19 infections might only be manifested as cognitive or functional decline, especially in patients with pre-existing cognitive disorders (185). Additionally, elderly patients with COVID-19 may only have fatigue, myalgia, headache, or digestive symptoms, including anorexia, vomiting without fever, or cough (37, 182, 183). Therefore, atypical presentations of SARS-CoV-2 infection in elderly patients are frequent and may delay diagnosis of the disease. Clinical care providers for elderly patients should be aware of these non-classical presentations. Furthermore, elderly COVID-19 patients tend to have more in-hospital complications when compared to younger populations (37). A multicenter retrospective study carried out in Hunan Province in China found that about 40% of elderly patients had complications such as acute respiratory distress syndrome, acute cardiac injury, acute kidney injury, sepsis and pneumothorax in comparison to 14.1% in younger patients (182). Moreover, more elderly patients received invasive mechanical ventilation compared to younger patients (182).

The underlying pathophysiology that leads to the worst outcomes in the elderly is poorly understood but probable contributing factors are waning immunosenescence and aging-related low-level proinflammatory responses leading to a gradual decline in the immune response creating a susceptible host.

Several mechanisms have been postulated to contribute to the observed higher risk of severe COVID-19 in the elderly (186), including: (a) increase in co-morbidities especially hypertension, diabetes, obesity and respiratory diseases; (b) the atypical presentation of illness or lack of any specific symptoms of the underlying COVID-19 in the elderly may delay the diagnosis and early management of these patients; (c) with aging, there is a decrease in the innate immune response, and a shift in T-cell subpopulations leading to a decrease in naïve T-cells and an increase in memory T-cells, which limits the response against novel infectious agents (186–190); and (d) in the elderly public health measures and quarantining of infected individuals might not be an easy task, especially with cognitively impaired patients (191, 192).

## COVID-19 Therapeutics

In the absence of effective drugs with established therapeutic efficacy against SARS-CoV-2, considerable attention has been focused on re-purposing currently available drugs for the treatment of COVID-19 (193). Drug trials have included anti-viral agents that were primarily licensed or developed for the treatment of influenza (oseltamivir, favipavir, umifenovir), HIV (lopinavir, ritonavir, azvudine), hepatitis (ribavirin, sofosbuvir), Ebola (remdesivir), herpetic infections (penciclovir), antibiotics with antiviral or anti-inflammatory properties (azithromycin), anti-parasitic including anti-malarial drugs (chloroquine, hydroxychloroquine, ivermectin), and the histamine-2 antagonist with anti-viral activities (famotidine). To circumvent or augment immune responses, several immunomodulatory drugs have also been studied including steroids, interferons, colchicine, interleukin inhibitors (tocilizumab, anakinra) as well as convalescent plasma. Therapies specifically directed at SARS-CoV-2 include the use of recombinant human ACE2 proteins to act as decoy receptors for the SARS-CoV-2 virus. An indication of the global research effort is reflected by a report stating that there are over 2,000 clinical trials assessing over 700 potential COVID-19 therapies (194). However, a “living” systematic review in the British Medical Journal concluded that: “*the effectiveness of most interventions is uncertain because most of the randomized controlled studies so far have been small and have important study limitations*” (195) This conclusion has been re-emphasized by Tikkinen et al. (196) stating: “*several false claims of efficacy have emerged from non-randomized comparisons–*.” In contrast, reports from the RECOVERY (Randomized Evaluation of Covid-19 Therapy trial) Collaborative group in the U.K. have established the benefits of a 28-days treatment with the steroid dexamethasone in reducing mortality and progression to ventilation support in patients with COVID-19; in contrast, no benefits were associated with the combination of lopinavir plus ritonavir (197).

### Chloroquine and Hydroxychloroquine

The initial interest in chloroquine and hydroxychloroquine was understandable as following the SARS epidemic in 2002, data from *in vitro* studies suggested that the drugs possessed both anti-viral and anti-inflammatory properties linked to their ability to raise endosomal pH in the host cell and inhibit glycosylation of the host ACE2 (Figure 3A) (198); a conclusion supported by data from China (88), but later challenged by data from *in vivo* studies with macaque monkeys (199). *In vitro* studies also suggest that chloroquine and hydroxychloroquine could block the binding of the virus to ACE2; however, the concentrations required would most likely be toxic if used clinically and would raise the risk of cardiac dysrhythmias (200). Furthermore, hydroxychloroquine and chloroquine have been extensively used for treating malaria, lupus erythematosus and rheumatoid arthritis and since their pharmacokinetic and toxicity profiles were well-known their use was considered safe. Unfortunately, inadequacies in the design of some of the clinical trials investigating these drugs in COVID-19 have resulted in contradictory results. In March 2020 clinical support for the effectiveness of a combination of hydroxychloroquine with the macrolide antibiotic, azithromycin, in reducing the viral load of SAR-CoV-2 in patients was acclaimed and widely publicized following an open label non-RCT study from Marseille (201). However, the small number of patients with a questionable recruitment process together with non-congruent results raised questions as to interpretation of the data. Nevertheless, in the absence of alternative options many global healthcare authorities endorsed use of hydroxychloroquine, including the FDA with the latter approving Early Use Authorization for hydroxychloroquine on March 28th 2020 (202). Subsequently, randomized control trials from Canada and the USA failed to demonstrate a post-exposure prophylaxis benefit (203). Similarly, a multicenter, open label randomized controlled study in Brazil using hydroxychloroquine and azithromycin not only failed to demonstrate beneficial outcomes but also reported adverse events including cardiac and hepatic toxicities (204). To complicate the scientific debate, retrospective studies produced conflicting results with one reporting no benefits of hydroxychloroquine or azithromycin alone, or in combination, to reduce in-hospital mortality (205), and another reporting a reduction in mortality with hydroxychloroquine alone or in combination with azithromycin versus standard of care (SOC) (206). In the latter study a far greater number of patients treated with hydroxychloroquine and/or azithromycin also received steroids, a confounding Achilles heel weakness apparent in retrospective studies (37). In late April 2020, the FDA reversed its approval and cautiously advised against the use of hydroxychloroquine or chloroquine outside clinical trial or hospital settings. Finally, data from the RECOVERY trial which is by far the largest RCT to date, annulled the effectiveness of hydroxychloroquine concluding: “*In patients hospitalized with COVID-19, hydroxychloroquine was not associated with reductions in 28-days mortality but was associated with an increased length of hospital stay and increased risk of progressing to invasive mechanical ventilation or death”* (207). Despite the evidence against the use of chloroquine or hydroxychloroquine the controversy continues. A meta-analysis that was posted on September 30th 2020 on the medRxiv preprint server concluded that hydroxychloroquine use in outpatients reduced hospital stay and mortality despite none of the five RCTs included in the analysis individually demonstrating benefit (208).

### Remdesivir

The anti-viral drug, remdesivir was originally developed for hepatitis C and later tested for Ebola and the pharmacokinetic and toxicity profile for this drug are also established albeit limited to data from small RCTs. Remdesivir is a pro-drug that readily enters cells where it undergoes hydrolysis to an active form by esterase enzymes to a triphosphorylated adenosine analog that competitively inhibits RNA-dependent polymerase resulting in termination of replication chain (Figure 3A). *In vitro* results indicate that the anti-viral activity of remdesivir is effective at sub, or low micromolar concentrations against other coronaviruses including MERS-CoV and subsequently supported by an *in vivo* study in Rhesus monkeys (209, 210). A multi-center RCT from China (211) compared the clinical outcomes of 158 with severe COVID-19 treated for 28 days with remdesivir against 78 patients provided with SOC. The authors concluded: “*In this study of adult patients admitted to hospital for severe COVID-19, remdesivir was not associated with statistically significant clinical benefits.”* The study also failed to demonstrate that remdesivir reduced the viral load. The results from an international multi-center study (ACTT-1) of hospitalized COVID-19 patients demonstrated that remdesivir in comparison to placebo shortened the time for recovery by 5 days, but did not reduce mortality, in adults hospitalized with COVID-19 and evidence of lower respiratory infection (212). Conversely, a study comparing 5 vs. a 10-days remdesivir treatment, but lacking a matching comparator SOC arm, found no difference in outcomes (213). Results, from a third RCT demonstrated a modest benefit for a 5-days management course but intriguingly not for a 10-days course (214). Finally, the interim results from the WHO SOLIDARITY multi-center international RCT indicate no benefits of remdesivir, hydroxychloroquine, lopinavir or interferon on overall mortality or initiation of ventilation and duration of hospital stay (215). Critically, RNA viral load was not measured to reflect anti-viral efficacy. In conclusion, the results from the clinical trials with remdesivir do not show that it reduces viral load or mortality rates although there is evidence from some but not all studies that patients recovered more rapidly.

### Dexamethasone

Although glucocorticoids, such as dexamethasone, will suppress the host's immune response and increase the risk of enhancing viral replication and potentially activate a latent infection such as tuberculosis or hepatitis they can be beneficial to counteract a hyperinflammatory cytokine storm as seen in severely ill COVID-19 patients (Figure 3B). The results of a RCT from the UK based RECOVERY study group demonstrated significant efficacy for the use of dexamethasone (207).

### Ivermectin, 3CLpro and PLpro Inhibitors

Another promising drug target is cysteine protease, Mpro (or 3CLpro), a precursor polyprotein, which is essential for viral cleavage (216). There is considerable interest in the potential antiviral benefits of the widely used broad-spectrum anti-parasitic drug ivermectin as a putative inhibitor the importin α/β1 protein complex preventing nuclear import and also inhibit the 3CLpro protease thus preventing viral replication (217–219). There is an extensive literature that supports the safety of ivermectin to treat a variety of parasitic infections as well as *in vitro* and *in vivo* data reflecting anti-SARS-CoV-2 effects (220). Although not receiving FDA approval for COVID-19 disease, ivermectin has been used for the treatment of the disease in a number of countries and notably in Peru where its use has been linked to a decrease in both mortality and lethality although in the absence of data from appropriate RCTs other non-drug related factors may have contributed to the apparent benefits of treatment (221, 222). An exploratory Phase 1 clinical trial has been approved in the U.K. by the Medicines & Healthcare products Regulatory Agency) to determine the therapeutic efficacy of ivermectin as a low cost prophylactic to prevent the spread of COVID-19 (223). Several other 3CLpro inhibitors are also being investigated including GC376, and its analog GC373, which are currently used to treat the fatal feline infectious peritonitis caused by a coronavirus. Both GC376 and GC373 have been shown to be very potent inhibitors of SARS-CoV-2 in a cell culture protocol using monkey and human lung cells infected with SARS-CoV-2. The drugs were effective with IC_50_ values in the nano-molar range with no apparent toxicity (224). Another target is the papain-like protease, PLpro, which plays an important role in dampening the host's inflammation and antiviral signaling pathways including inhibition of type I interferon production (225).

### Famotidine

Famotidine is a histamine receptor 2 (H2) antagonist that is widely used as an antacid and *in vitro* studies indicate that the drug has inhibitory effects on several viral proteases including 3CLpro, PLpro and TMPRSS2 (226). Retrospective clinical data are supportive of the benefits of famotidine in reducing mortality in COVID-19 patients independent of its gastric acid lowering action as indicated by the lack of benefits associated with the use of proton pump inhibitors (227). Data from a number of Phase III clinical trials with famotidine are expected in 2021.

### TMPRSS2 and Furin Inhibitors

In addition, there are several experimental drugs, which appear promising as potential serine protease inhibitors that target the two host enzymes, TMPRSS2 and furin that are hijacked by the SARS-CoV-2 virus, to facilitate binding to ACE2 and entering the host cell (228–230). Camostat mesilate, a drug approved in Japan for use in pancreatic inflammation, and the related nafamostat, have also been shown in *in vitro* studies to inhibit TMPRSS2, with IC50s of 142 and 55 nM, respectively (231). Camostat is undergoing clinical trials for the treatment of COVID-19 with one with the Identifier NCT04608266 now in Phase III “*A Multicenter Randomized Trial to Evaluate the Efficacy and Safety of Camostat Mesylate for the Treatment of SARS-CoV-2 Infection—COVID-19 in Ambulatory Adult Patients (CAMOVID).”* Worthy of note is that because TMPRSS2 and furin are important for a variety of cellular processes in the host the risk of side effects may be heightened with the chronic use of inhibitors of these enzymes.

### Convalescent Plasma and Targeted Antibody Therapy

As previously mentioned the clinical data supporting the use of convalescent plasma therapy for treating COVID-19 patients has not demonstrated positive benefits (141); however, progress has been made in the use of synthetic antibodies to treat COVID-19 patients. Eli-Lilly received EUA from the FDA for their single antibody, bamlanivimab (LY-CoV555) on the 9th November 2020 and on the 21st November the biotechnology company Regeneron also received EUA for their product, REGN-CoV2. LY-CoV555 is a single highly potent antibody isolated from convalescent COVID-19 patients that binds to the RBD of the virus spike protein (232). Regeneron's antibody cocktail consists of two antibodies, casirivmab (REGN10933) and imdevimab (REGN10987) that also target and irreversibly bind to the RBD site on the SARS-CoV-2 spike protein and was also based on screening human neutralizing antibodies from recovered COVID-19 patients (233). For both LY-CoV555 and REGN-CoV2 positive data has been published indicating that the antibody cocktails are particularly effective in patients with high viral loads or whose immune system had not responded to the virus, however, they are not effective in severely ill hospitalized patients (103, 234, 235). AstraZeneca is also developing an antibody targeted therapy, AZD7442, for the prophylactic treatment of adults exposed to COVID-19 with data from a Phase III clinical trial, NCT04507256, expected in 2021 (236).

## Vaccine Progress

When compared with other epidemic viruses, SARS-CoV-2 is unique as it is highly contagious, can be transmitted easily even before development of clinical symptoms and has been associated with significant morbidity and mortality. Although the virus has spread to all continents, the total number of those infected (~2%) is well below estimated need for herd immunity (~>70%) hence there is urgency for the development of effective vaccines. The benefits of effective vaccines are well-established as has been well-documented for other historic pandemics (237). The global acceptance of effective vaccines will allow for relaxing restrictive measures and recovery of socioeconomic conditions as well as reversal of the psychological consequences associated with a pandemic. There has been a global response to developing vaccines and its acceptance, and an up to date status can be found through accessing vaccine tracker sites (238).

In April 2020 the US government launched Operation Warp Speed (OWS) with the objective of delivering at least 300 million doses of a safe and effective vaccine by January 2021 (239). The process involves collaboration between several government agencies including the Department of Health and Human Services (HHS), the Centers for Disease Control and Prevention (CDC), the Food and Drug Administration (FDA), the National Institutes of Health (NIH), and the Biomedical Advanced Research and Development Authority (BARDA), as well as the Department of Defense (DoD). However, there are concerns regarding OWS and its potential to damage international efforts to make effective vaccines globally available and this is evident by the exclusion of cooperation with the WHO, the European Commission, and the Coalition for Epidemic Preparedness Innovations (CEPI) as well as exclusion of vaccines from China. CEPI, based in Norway, was launched in 2017 with co-funding by the Bill and Melinda Gates Foundation, the Welcome Trust and a consortium of nations and is recognized as a key player in the development of vaccines against potential epidemic threats (240).

### Leading Potential Vaccines

A comparison of the leading potential and approved vaccine platforms for SARS-CoV-2 can be found at (241, 242) and are summarized in Table 1.

**Table 1:** Details of prime potential or approved COVID-19 vaccines and their latest stages of development.

**Vaccine platform**	**Candidate vaccine**	**Mechanism**	**Research institute**	**Planned route; doses**	**Published phase 1 & 2 results**	**Status of phase 3 clinical trials**
Recombinant protein particles vaccines	NVX-CoV2373	Deployment of full length recombinant viral spike protein with Matrix M adjuvant	Novavax, USA	IM; Two: day 0 and day 21	(243)	Completed enrollment, results expected March 2021
Inactivated	Three inactivated vaccines	Whole virus propagated into cell culture line then inactivated with organic compounds. Purified material adsorbed with aluminum adjuvant	Sinopharm, Wuhan/Beijing Institute of Biological Products, China	IM; Three: day 0, day 28, and day 56	(244)	Started Jul 2020, No published results Approved for emergency use
Vector based	ChAdOx1-S/AZD1222	Deployment of spike protein through replication deficient Chimpanzee adeno virus	University of Oxford/Astra Zeneca, UK	IM; Two day 0 and 28	(245–247)	Published Dec 2020 Approved for emergency use
	Ad5-vectored COVID-19	Deployment of spike protein through replication deficient type five adeno virus	CanSino Biological Institute/Beijing Institute of Biotechnology, China	IM; Single	(248)	Started June 2020 No published results Approved for emergency use
	Gam-COVID-Vac (Sputnik V)	Deployment of spike protein through replication deficient type 5 and 26 adeno viruses	Gamaleya Research Institute, Russia	IM; Two: day 0 and day 21	(249)	Started Sep 2020 No published results Approved for emergency use
mRNA	mRNA−127	Nanoparticle formulated mRNA encoding spike protein or receptors binging proteins	Moderna, USA	IM; Two: day 0 and 28	(250, 251)	Published Dec 2020 Approved for emergency use
	BNT162b1		BioNTech/Fosun/Pfizer, Germany, USA	IM; Two: day 0 and day 21	(252, 253)	Published Dec 2020 Approved for emergency use

*IM, Intramuscular*.

#### Viral Vectors Vaccines

The concept of viral vector vaccines is based upon deployment of viral particles such as the SARS-CoV-2 S spike attachment proteins and other similar proteins through a suitable replication deficient vector such as human or Chimpanzee adeno or influenza viruses to produce an appropriate immune response. The approach has been researched for several years and has demonstrated preliminary success against Dengue virus, Ebola virus disease, Zika virus and candidate vaccines for MERS-CoV (254). However, pre-existing immunity from previous related viral infections has shown to diminish appropriate responses (255).

Four vector vaccines are at different advanced level of efficacy evaluation or granted emergency approval by licensing authorities at different countries:

1. CanSino Biologics (Ad5-nCoV) from China, which utilizes replication deficient human adeno virus-5 as a vector in a single dose (248). Ad5-nCoV has demonstrated immunogenic responses in Phase I/II trials with good safety profiles and has been released for Phase III trials in China, the Middle East and South America. In August 2020 Chinese authorities granted accelerated vaccine patency and approval.
2. Gamaleya (Sputnik V vaccine) from Russia, which uniquely deploys dual replication deficient recombinant human adenoviruses (Ad5 and Ad26) for immune evasion that requires two doses. The Sputnik V vaccine has been granted early approval after the published results indicate combined vaccine immunogenicity profiles particularly following the second dose, as well as a positive safety profile (249, 256).
3. University of Oxford/AstraZeneca (ChAdOx1 nCoV-19) vaccine, which utilizes replication deficient Chimpanzee adenovirus for immune evasion. ChAdOx1 nCoV-19 has received considerable interest from many healthcare authorities since it was believed to be ahead of other vaccines based upon earlier progress as a candidate for the related MERS-CoV (245). In May 2020, ChAdOx1 nCoV-19 vaccine became the first vaccine to be tried against COVID-19 in humans and has demonstrated favorable immunogenic and safety profiles in Phase I/II trials. ChAdOx1 nCoV-19 entered Phase III trials in July 2020, and Phase II/III data published from randomized controlled studies in Brazil, South Africa and UK published in December 2020 (238). Due to a dosing error in the UK study some vaccine vials were filled with half the intended dose and the protocol was changed so as to compare people who received a half dose followed by the full dose with those given two full doses. The results were interesting as the effectiveness was higher, 90%, for those who first received a half dose vs. who received the full dose for both injections, 62%, which intriguingly raises the possibility that the full first dose vector mediated immunogenicity, which might hampered efficacy of the second dose. The observation certainly caught the attention of the trial investigators and it will be pursued with additional studies.

On December 30th 2020, ChAdOx1nCoV-19 was approved for use in the UK for use in 18 years or older An advantage of the Oxford/AstraZeneca vaccine vs. the mRNA vaccines is its stability at regular refrigerator temperature and relatively low cost agreed with manufacturer at $2 to $3 USD/dose (257, 258).

In December 2020, AstraZeneca announced collaboration with Gamaleya to determine if the appropriate combination of ChAdOx1nCoV-19 with the Sputnik V vaccine will further boost effectiveness (259).

Ad26.CoV2-S produced by Johnson and Johnson Pharmaceuticals is a non-replicating adenovirus serotype 26 vector- based vaccine expressing the stabilized pre-fusion spike (S) protein of SARS-CoV-2, initially planned as a single dose formulation, but now with a trial as a two-dose formulation (238). Data from a Phase 1/2 clinical study demonstrated that the safety profile and immunogenicity after a single vaccination were very encouraging which allowed the investigators to move to a Phase 3 clinical trial (238).

#### Inactivated Virus Vaccines

Based on a well-established vaccine methodology, inactivated virus technique is simple and attractive. In China Sinovac/Instituto Butantan have reported success of PhaseI/II trials and their vaccine, CoronVac, entered Phase III studies in Brazil, Indonesia, and Turkey using an inactivated vaccine cultured in Vero cell line, inactivated with inorganic compounds then combined with an aluminum adjunct (244, 260). A limitation is that there are, to date, no phase III data published in peer-reviewed journals. However, media reports from Turkey stated the vaccine's seroconversion is as high as 97%, though this does not refer to its effectiveness, and preliminary data from a trial in Brazil stated, according to a media release on January 8th, 2021, effectiveness of 78%, but subsequently revised to 50.4% (261).

Similarly, the Beijing Institute of Biological Products developed BBIBP-CorV, which is also based on an inactivated virus platform as for CoronaVac. This vaccine has undergone clinical trials through the state-owned SinoPharm and received emergency use in China in mid-2020 and administered to >20 million Chinese. In addition, as a result of Phase III trial data effectiveness is reported to be between 79 and 86% and in September 2020 the vaccine was provided to health care workers in UEA and became fully available to the public in early December 2020 (262).

#### Recombinant Technology

In the USA Novavax (NVX-CoV2373) has developed a nanoparticle vaccine based on the SARS-CoV-2 spike glycoprotein recombinant technology with Matrix-M1 immunogenic adjuvant and favorable efficacy and safety profiles have been published (243). Integral to the vaccine's technology are the advantages of the capability for mass production, relatively cheap costs, together with ease of storage; all crucial requirements for ideal vaccine development. A randomized clinical trial to assess efficacy has been completed in the UK with enrollment of 15,000 patients and plans to publish the results in the first quarter of 2021. Additional clinical trials are also underway in Mexico and South Africa (263).

#### mRNA Vaccines

In recent years, the use of nucleic acid-based technology for vaccine production has developed rapidly and has overcome previous delivery problems by utilizing encapsulated lipids nanoparticles (264, 265). Once the gene sequence is known a nucleic acid-based vaccine can be developed rapidly and subject to the success of clinical tests could be available for use within a year; a potential issue is the need for low temperature storage, which for BioNTech/Fosun/Pfizer (BNT162) is −70C. In the case of COVID-19 an mRNA vaccine would provide the genetic information, and not directly the protein, to produce the spike protein to which the host now makes protective antibodies.

Progress with the mRNA 1273 mRNA-based vaccine was rapid, and it demonstrated favorable immune responses both in younger and older patients without significant adverse events (250, 266, 267). Similarly, BNT 162 completed Phase1 studies and entered a Phase II/III safety and efficacy evaluation with preliminary data released on November 9th 2020. Phase III data published in late 2020 indicate that both the Moderna/NIAID (mRNA1273) and BioNTech/Fosum/Pfizer (BNT162) vaccines demonstrate >90% efficacy at preventing COVID-19 and with only transient local and systemic adverse reactions (251–253, 268, 269). Both vaccines have received EUA in several countries and the first person to be vaccinated with the mRNA vaccine, BNT162, was a 90-years old woman in the United Kingdom on December 8th, 2020). The mRNA vaccines may prove to be more expensive than others because of the cost of the advanced technology and for Moderrna's vaccine, which has already been priced at $74.00 USD for a full two-dose course (270). Although not evident from the Phase III studies several severe allergic responses have been reported for the BNT162 vaccine in people who had a previous history of allergies and may be attributed to the use of polyethylene glycol in the packaging of the lipid nanoparticles (271).

### Other Potential Vaccines

Included in the international efforts to develop a vaccine for COVID-19 is a team at Imperial College, London, who are developing a self-amplifying RNA vaccine that theoretically should provide greater efficacy than other mRNA vaccines and is in Phase I/II studies (272).

## Comparison of How Countries Have Responded to Covid-19 and Preparing for Future Pandemics

In the absence of an effective vaccine or drugs with proven effectiveness against SARS-CoV-2 other NPIs, including quarantine, track and trace technologies, and mandatory mask wearing, have been variably used by countries with vastly different effectiveness. We briefly review these issues and discuss how the lessons learnt can help prepare the world for future pandemics.

The level of preparedness of countries to deal with a major health emergency such as a pandemic was reviewed in 2019 in the Global Health Security (GHS) report (ghsindex.org). The GHS ranked 195 countries in six categories: Prevention; Detection and Reporting; Rapid Response; Health System; Compliance with International Norms; and Risk Environment. The USA and the UK were ranked #1 and #2, respectively for demonstrating highest level of preparedness. In terms of Asian countries only Thailand (#6), South Korea (#9), and Malaysia (#18) appeared in the top 20 with Vietnam ranked #50, and China #51; Taiwan was not included. When these rankings are compared to the numbers of COVID-19 infections and deaths per their respective population size it is clear that the GHS rankings do not correlate well with the response to COVID-19 (1). Thailand, South Korea and Malaysia initially managed to keep the rate of infections and deaths relatively low compared to the other aforementioned countries and Vietnam fared even better and their successful strategy can be credited to its experience in 2003 with SARS-CoV and best summarized as “*Survival of the Smartest”* (273). After the first COVID-19 patient was confirmed on January 21st 2020 the Taiwan CDC launched a proactive campaign to investigate and track and trace contacts and as of November 3rd has kept deaths <10 per million population (274). Eliminating or maintaining a low level of infections has proved difficult for some countries and a retrospective analysis of how different countries have fared will be valuable for planning for future pandemics. Of particular importance, as discussed by Haug et al. (275), will be determining which NPIs are effective and at the same time minimize negative impacts on the health of the public and economy. Thus, whereas lockdowns, curfews, restrictions on travel and gatherings including school closures, imposing work from home policies are among the most effective measures there are also negative consequences such as negative effects on the education and social development of children, domestic violence as well-neglecting the follow up of patients with chronic diseases such as cancer and major effects on the economy (275). Evidence of the value of lockdowns to contain the spread of the virus was provided by New York State, which was initially hit very hard by COVID-19 pandemic, but the subsequent lockdown resulted in an estimated >50% reduction in transmission for all age groups and an ~7% overall reduction when the wearing of a facemask was required (276).

The “*Independent Panel for Pandemic Preparedness and Response,”* consisting of 13 health experts and former political leaders has been established by the WHO to review the response to COVID-19 in its member countries; the report will strive to provide useful insights and aid in planning for future pandemics. Meanwhile, *post-hoc* analysis has started with a report in The Lancet on September 24th that compared how five countries in Asia (Hong Kong, Japan, New Zealand, Singapore and South Korea) vs. 4 in Europe (Germany, Norway, Spain and UK) responded to the challenges of easing COVID-19 restrictions through until mid-June 2020 (277). The report analyzed six approaches: Overall Strategy; Knowledge of infection status; Community Engagement; Public-Health Capacity; Health-System Capacity, and finally, Measure for Border Control. One of the universally identified bottlenecks in mounting a rapid response was the frailty of the medical supply chain.

Amongst the confusing messages that may have perpetuated the spread COVID-19 was the question of the benefits of the use of facemasks and the importance of aerosol spread; the latter being particularly important in enclosed areas with inadequate ventilation. Some countries recognized the benefits and made the use of facemasks mandatory whereas others dithered and in consequence public acceptance was compromised (278). Unfortunately, there are only a limited number of studies that have rigorously investigated the effectiveness of different types of masks (279). Of interest, but requiring further study is evidence presented in The New England Journal of Medicine, albeit speculative, suggests that wearing a mask may help develop immunity via variolation (280, 281).

One year after SARS-CoV-2 was first identified, questions still remain concerning the pathophysiology of COVID-19. Especially problematic is the persistence of symptoms in patients in the absence of a positive COVID-19 test; the so-called “*lasting misery of coronavirus long haulers”* with an estimate that 10% of those infected with SARS-Co-2, may be facing long-term pulmonary and cardiovascular and other disabilities (281). In addition, the identification of cases of reinfection raises the question of how long immunity will last (282). On the other hand, we have gained considerable knowledge about optimizing supportive care for COVID-19 patients including the use of prone positioning for those with moderate to severe respiratory distress (283).

Many European countries, United States, Canada, India, South Africa and Brazil saw a resurgence in cases during the autumn/winter of 2020, and health authorities in these countries brought back lockdowns and other restrictions to curb infections. Several reasons can be offered to explain the spike in COVID-19 cases in these countries, among these are (a) countries in the Northern hemisphere did not see a summer increase in COVID-19 cases, but when the temperature started to cool in the autumn and winter people spent more time indoors, less social distancing and increasing the risk of viral transmission; (b) Covid-fatigue caused people to lapse in their use of NPIs and lack of vigilance at home and away; (c) the re-opening of schools also may have contributed to the increase in transmission and spread by asymptomatic children (284); and (d) increases in the transmission rate, which has been attributed to the emergence of new SARS-CoV-2 variants such as B.1.1.7 in the United Kingdom and 501.v2 in South Africa (68, 285).

The WHO has warned that the virus will continue to spread rapidly in 2021 (34). Several countries have approved coronavirus vaccines for use but as populations await their rollout, cases remain high across many parts of the world (286).

## Conclusion

Global collaborative efforts are required for better control of the current pandemic and to plan for future pandemics. A team at University College London has developed a computer-modeling program that uses data about societal impact on the environment, including climate and changes in animal habitat, to predict where future zoonotic pandemics might emerge (287). This approach successfully predicted the origins, but not the specific timing, of the Ebola outbreak (288). “*Global Virome”* has a scientific board comprised of leaders in the fields of global health and pandemic prevention with the goal of global collaboration via the “*Trinity Challenge”* initiative (289). Morens and Fauci (290) remind us that pandemics are not new and they discuss the impact of re-emerging/resurging pandemics such as tuberculosis, SARS-CoV, MERS-CoV, and SARS-CoV-2. Vidal (287) and Morens and Fauci (290) emphasize the negative effects that humans have on the environment and inevitably we must expect “backlashes” from nature.

## Author Contributions

AS wrote the manuscript with substantial input from all the authors HD, CT, and HH therapeutics and vaccines. HH pathophysiology. MI figure. CT, DB, HD, EF, HH, MI, and AS provided critical feedback to improve the manuscript. All authors contributed to the table, literature search, contributed to the article, and approved the submitted version.

## Conflict of Interest

The authors declare that the research was conducted in the absence of any commercial or financial relationships that could be construed as a potential conflict of interest.
